# Complete nucleotide sequence and annotation of the temperate corynephage *ϕ*16 genome

**DOI:** 10.1007/s00705-017-3383-4

**Published:** 2017-04-28

**Authors:** Juliya S. Lobanova, Evgueni R. Gak, Irina G. Andreeva, Konstantin V. Rybak, Alexander A. Krylov, Sergey V. Mashko

**Affiliations:** Ajinomoto-Genetika Research Institute, 1st Dorozhny pr. 1-1, Moscow, 117545 Russian Federation

## Abstract

The complete genome of *ϕ*16, a temperate corynephage from *Corynebacterium glutamicum* ATCC 21792, was sequenced and annotated (GenBank: KY250482). The electron microscopy study of *ϕ*16 virion confirmed that it belongs to the family *Siphoviridae*. The *ϕ*16 genome consists of a linear double-stranded DNA molecule of 58,200 bp (G+C = 52.2%) with protruding cohesive 3’-ends of 14 nt. Four major structural proteins were separated by SDS-PAGE and identified by peptide mass fingerprinting technique. Using bioinformatics analysis, 101 putative ORFs and 5 tRNA genes were predicted. Only 27 putative gene products could be assigned to known biological functions. The *ϕ*16 genome was divided into functional modules. Seven putative promoters and eight putative unidirectional intrinsic terminators were predicted. One site of putative «-1» programmed ribosomal frameshifting was proposed in the phage tail assembly genome region. *C. glutamicum* genetic tools could be broadened by exploiting the known integrase gene (gp33) and the newly identified excisionase gene (gp47), participating in site-specific recombination between *ϕ*16-*attP*/*attB*.

**Electronic supplementary material:**

The online version of this article (doi:10.1007/s00705-017-3383-4) contains supplementary material, which is available to authorized users.

*Corynebacterium glutamicum* is widely used to produce commercially interesting bio-based substances [1]. Phages present a problem for the biotechnology industry and cause financial losses. Many corynephages have been isolated, but only a few of them have been completely sequenced (*e.g.* [2, 3]). In the present study, the genome of *ϕ*16, a temperate corynephage from *C. glutamicum* (ATCC 21792), kindly provided by Dr. Trautwetter [4], was sequenced and annotated. This information could provide valuable evolutionary insights and be helpful for phage-resistant strain construction [5]. Different integrative vectors targeting different *attB*-sites have been constructed based on known integrases of phages *ϕ*AAU2 [6], beta [7], *ϕ*304L [8] and *ϕ*16 [9] from *C. glutamicum* strains. The newly identified *ϕ*16 excisionase, in addition to the known integrase gene, could be useful for broadening *C. glutamicum* genetic tools, *e.g.* for site-specific integration/excision of DNA fragments into bacterial chromosomes, as was demonstrated for other phage-based systems [10].

Phage *ϕ*16 was induced from the natural lysogen, *C. glutamicum* ATCC 21792, propagated and purified by centrifugation in CsCl-gradient as described [4, 11].

Transmission electron microscopy study of *ϕ*16 virion confirmed that it belongs to the family *Siphoviridae*, with a polyhedral head of 73 nm in width and 336 nm in length, and with a non-contractile striated tail of 14 nm in diameter (Fig. 1a), in line with Dr. Trautwetter’s group data [4]. Subsequently, one of the putative *ϕ*16 gene products (gp), gp16, was assigned to the tail tape measure protein (TMP). The relationship between observed tail length (~336 nm) and TMP size (2,151 aa), with a ratio of 0.156 nm/aa, is reasonable [12].

**Fig. 1:**
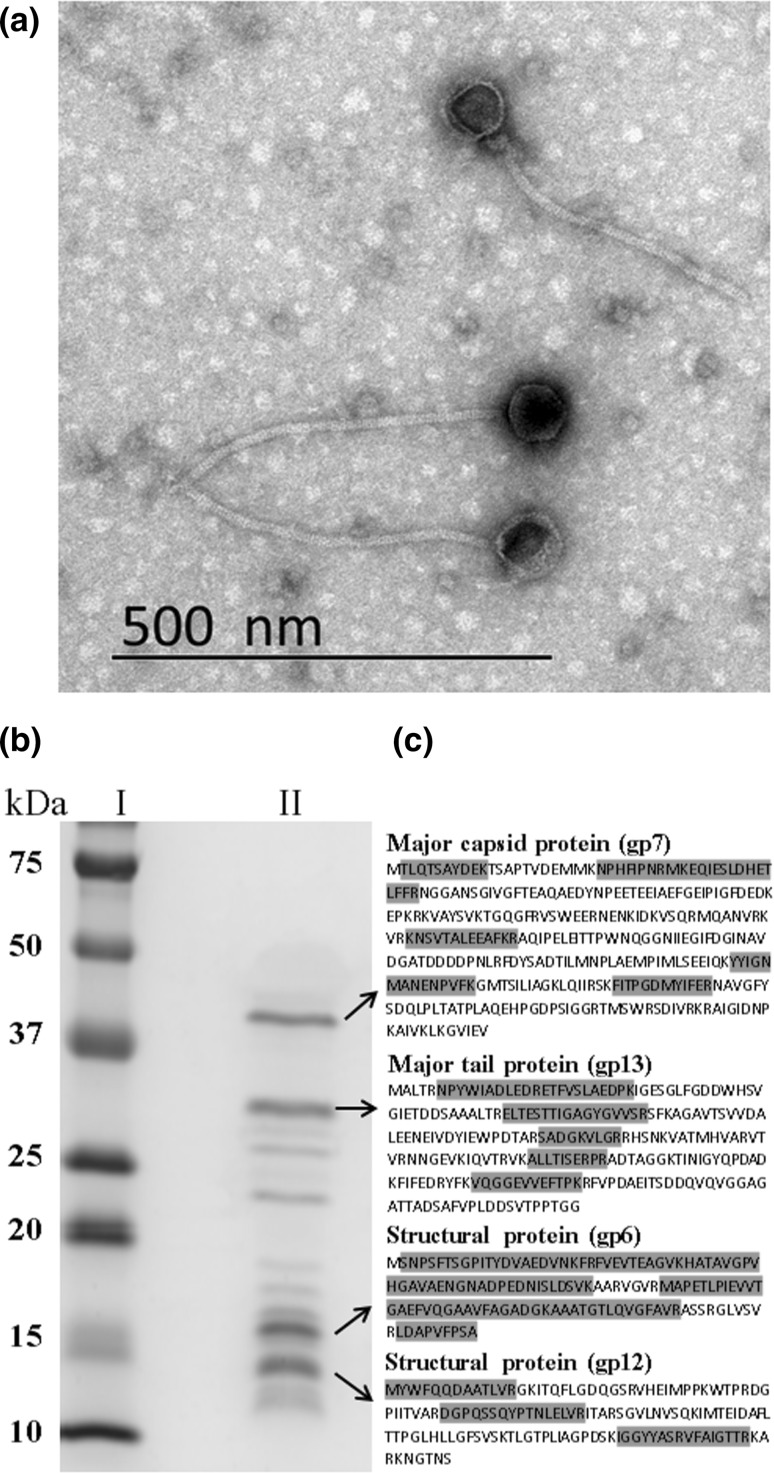
**(a)** Electron micrograph of *ϕ*16. Bar, 500 nm. **(b)** SDS-PAGE analysis of *ϕ*16 structural proteins. Molecular weight markers (lane I). Protein profile of *ϕ*16 (lane II). **(c)** Four major bands underwent peptide mass fingerprinting analysis; the corresponding predicted amino acid sequences (not highlighted) and the aa sequences detected in the analysis (highlighted) are shown

Purified phage DNA was hydrodynamically sheared, and fragments of 2 to 5 kb in size were blunted and then cloned and sequenced by the Sanger’s method. A total of 346 individual DNA fragments were sequenced with an average length of 750 ± 130 bp, and an achieved sequence coverage of ~ 6.6-fold. Closure of gaps was accomplished by primer walking. The genome sequence was finalized by determining the cos sequence with a sequence run-off experiment and comparison of the nucleotide sequence with the ligated phage ends.

Three bioinformatic on-line programs were used for ORFs prediction in the *ϕ*16 genome: Open Reading Frame Finder (https://www.ncbi.nlm.nih.gov/orffinder/), Glimmer 3 (http://www.ncbi.nlm.nih.gov/genomes/MICROBES/glimmer_3.cgi) and GeneMark S (http://exon.biology.gatech.edu/). The putative tRNAs genes were predicted using tRNAscan-SE (http://lowelab.ucsc.edu/tRNAscan-SE/). Two web services, the phiSITE PromoterHunter (http://www.phisite.org/main/index.php?nav=tools&nav_sel=hunter) (with parameters for “–10” and “–35” [Supplementary Fig. 1]) and the PePPER tool-box (http://pepper.molgenrug.nl) were used to search for putative promoters. Intrinsic terminators were identified with “ARNold finding terminators” (http://rna.igmors.u-psud.fr/toolbox/arnold/index.php) with additional evaluation of free energy parameters by the online version of the ViennaRNA package (http://rna.tbi.univie.ac.at/).

The *ϕ*16 genome is a double-stranded DNA molecule of 58,200 bp in length (G + C = 52.2%) with 3′-protruding single-stranded cohesive ends of 14 nt (3′-GGAAGGTGGAGGCT and CCTTCCACCTCCGA-3′). Using bioinformatics analysis, 101 putative ORFs covering ~92.4% of the total DNA length were identified. Only 27 gp(s) could be assigned to known biological functions (Supplementary Table 1); the other 55 gp(s) displayed homology to hypothetical proteins, and 19 gp(s) had no homologues in the databases (Fig. 2). Seven putative promoters (1 – leftward and 6 – rightward) and eight putative intrinsic unidirectional terminators (1 – leftward and 7 – rightward) were predicted in the intergenic spaces of the *ϕ*16 genome (Fig. 2, Supplementary Table 2, 3). Five tRNA Lys(UUU), Arg(UCU), Asn(GUU), Tyr(GUA) and Trp(CCA) were identified (Supplementary Fig. 2). Comparison between *ϕ*16 and *C. glutamicum* codons frequency support the hypothesis that phage-encoded tRNAs could compensate codon frequency bias and promote efficient translation of phage-derived mRNA [13] (Supplementary Fig. 3).

**Fig. 2:**
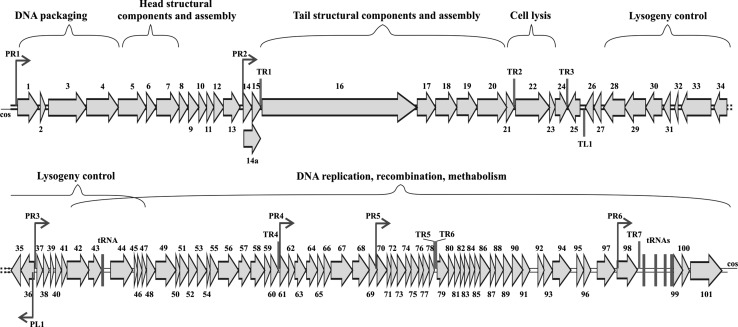
Genomic organization of *ϕ*16 phage. ORFs are numbered consecutively from left to right and are indicated by arrows (or triangles) in the direction of transcription. ORFs, joined by braces, are provided for the proposed functional modules of the *ϕ*16 genome. Promoter positions and directions are indicated by thin arrows; intrinsic terminators and tRNAs are depicted as dark and light boxes, respectively

Based on homology to known phage proteins, functional domains, and mutual arrangement, putative functions were assigned to products of 27 predicted ORFs (Supplementary Table 1). The entire genome was divided into the six functional modules (Fig. 2). The DNA packaging module contains small and large terminase subunits (gp2 and gp3) and the portal protein (gp4). The prohead protease (gp5), the major capsid and tail proteins (gp7 and gp13), the tail TMP (gp16), and the tail fiber protein (gp20) could be predicted in the structural components and assembly module. At the same time, four major structural proteins, the major capsid and tail proteins (gp7 and gp13) and two proteins with unknown function (gp6 and gp12), were detected by SDS-PAGE and identified by trypsin-based peptide mass fingerprinting technique (PMF), using Ultraflex II LC-MALDI-TOF/TOF (Brucker), performed according to Govorun et al. [14]. Furthermore, detection of N-terminal Met residue retention in a trypsin-digested peptide from gp12 and its elimination from N-terminal peptides from gp6 and gp7 confirmed the N-terminal processing rule [15] (Fig. 1b, c).

A putative site of a –1 programmed ribosomal frameshifting (PRF) could be found in the proposed tail assembly genes and was composed of three functional elements: an internal SD (5’–GAGG→3’), a “slippery sequence” (5′–GGGGGAA→3′) and an H-type pseudoknot RNA structure (Supplementary Fig. 4) [16]. The PRF was predicted to lead to the formation of a large fusion protein, gp14A.

Homologues of two known enzymes were predicted in the host lysis module: the endolysin (gp22) and the holin (gp23). The lysogeny control module was unusual: it contained two putative integrases (gp33 and gp28), the excisionase (gp47), the phage superinfection exclusion protein (gp34), and the transcriptional regulator (gp36). The nucleotide sequence of the *ϕ*16 *int* gene (corresponding to ORF33 in our annotation) and the *ϕ*16 *attP* site were deposited previously (GenBank: Y12471.1) [8] and differ from the newly sequenced ORF33 in several points due to sequencing errors in the past, that resulted in differences in the structures of the corresponding gp(s) (Supplementary Fig. 5). We confirmed the ability of gp33 to provide site-specific integration of recombinant DNA into the *ϕ*16-*attB* of the *C. glutamicum* ATCC 21792c chromosome, which was previously shown by the Trautwetter group [9]. We also demonstrated experimentally the effective excision of integrated recombinant DNA when gp33 and gp47 are expressed simultaneously of (manuscript in preparation). The experiments also showed that the second putative integrase, gp28, could not use the previously established *ϕ*16-*attP* site [8] for site-specific recombination. No other putative *attP*-site was detected in the vicinity of ORF28 (unpublished result).

The replication/recombination/metabolism module also contained homologues to known proteins: ParB-like protein (gp42), HNH homing endonuclease (gp52), the transcriptional regulator (gp56), SSB protein (gp58), glutaredoxin (gp62), RusA endodeoxyribonuclease (gp63), methyltransferase (gp64), chromosomal partitioning protein (gp68), oligoribonuclease (gp94) and ATPase (gp101).

The analysis indicated that some modules of the *ϕ*16 genome had complete or partial homology to distinct chromosomal regions of four bacteria, leading us to hyphothesize that these are uncharacterized prophages in bacterial genomes. Throughout large parts of the genome sequence, significant similarity was observed between *ϕ*16 and the hypothetical prophage *Corynebacterium pyruviciproducens* ATCC BAA-1742, at the nucleotide and deduced protein sequence level. Significant similarity was also observed, throughout the whole genome, between *ϕ*16 and the hypothetical prophage *Brevibacterium flavum* ATCC 15168 (identical to prophage of *C. glutamicum* ATCC 14067). We also observed similarity between predicted *ϕ*16 proteins, which are involved in DNA packaging, head and tail structural components, as well as assembly modules, and proteins of a *Corynebacterium ulcerans* BR-AD22 hypothetical prophage. Several *ϕ*16 lysogeny control genes (ORFs 30, 33, 35, 47) were very similar, at the protein and nucleotide level, to genes of the hypothetical prophage of *C. falsenii* DSM 44353, strain BL 8171. Furthermore, a significant part of the *ϕ*16 genome, as well as the second part of the lysogeny control genes (ORFs 28, 29, 34, 36) share homology with a hypothetical prophage from *C. pyruviciproducens* ATCC BAA-1742. To demonstrate the general sequence homology with the four hypothetical prophages, multi-dot plots were obtained (Supplementary Fig. 6). Neither protein nor nucleotide homology was observed between *ϕ*16 and BFK20 or P1201. However, the *ϕ*16 genome is not present in any bacterial chromosome in the database; therefore, the sequence of the entire *ϕ*16 phage genome was deposited for the first time in GenBank, under accession number KY250482.

## Electronic supplementary material

Below is the link to the electronic supplementary material.

Supplementary material 1 (DOC 981 kb)

Supplementary material 1 (TXT 140 kb)
