# Supplementary material for: Complete nucleotide sequence and annotation of the temperate corynephage ϕ16 genome
Source: Arch Virol. 2017 Apr 28;162(8):2489–92. doi: 10.1007/s00705-017-3383-4 (PMC5506516; doi:10.1007/s00705-017-3383-4)
Supplement: Supplementary file 1 — Supplementary material 1 (DOC 981 kb) [file 705_2017_3383_MOESM1_ESM.doc]

**Complete nucleotide sequence and annotation of the temperate corynephage *φ*16 genome**

Juliya S. Lobanova, Evgueni R. Gak, Irina G. Andreeva, Konstantin V. Rybak,

Alexander A. Krylov, Sergey V. Mashko

*Ajinomoto-Genetika Research Institute, 1st Dorozhny pr. 1-1, 117545 Moscow, Russian Federation*

E-mail addresses**:** Yulia_Lobanova@agri.ru (J. Lobanova), Evgueni_Gak@agri.ru (E. Gak),

Irina_Andreeva@agri.ru (I. Andreeva), Alex_Krylov@agri.ru (A. Krylov), Konstantin_Rybak@agri.ru (K. Rybak), Sergey_Mashko@agri.ru (S. Mashko)


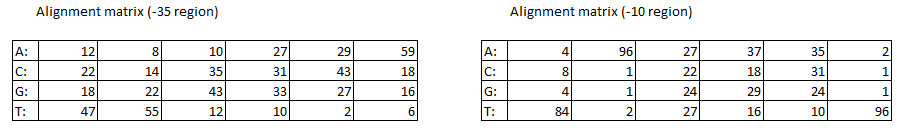


**Supplementary Fig. 1** The input alignment matrices for the phiSITE Promoter Hunter program for the “-10” and
“-35” motifs, calculated on the basis of data for *Corynebacterium glutamicum* housekeeping promoters, published by Pfeifer-Sancar et al.[[1]](#footnote-2)


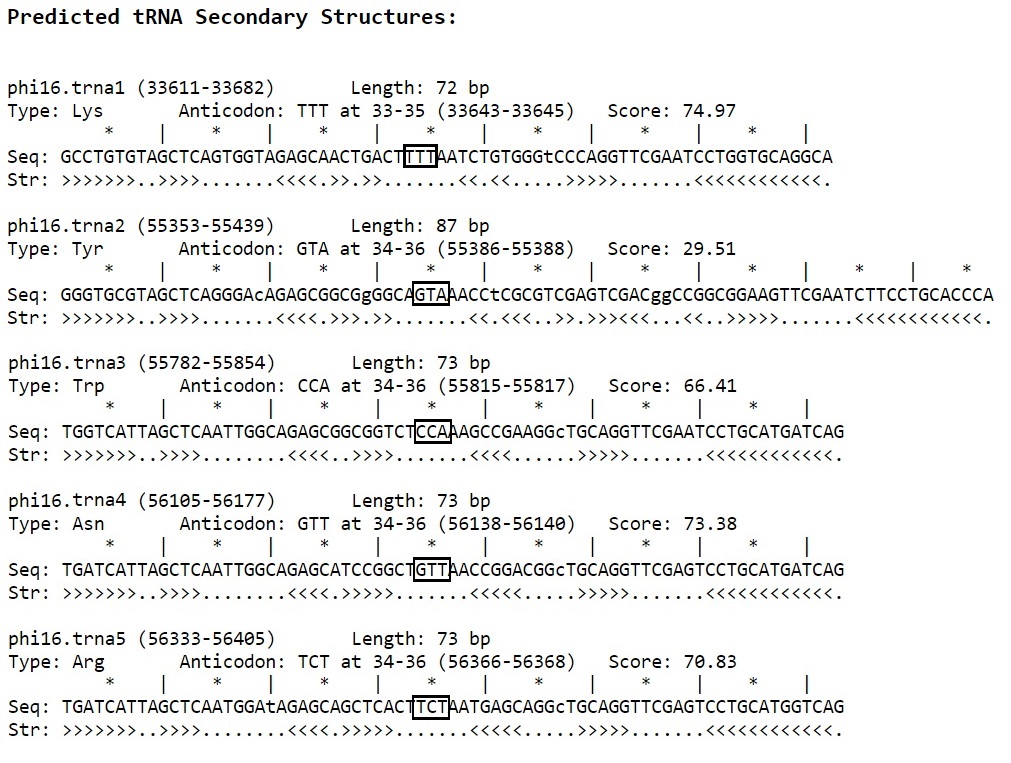


**Supplementary Fig. 2** Predicted tRNA secondary structure of *φ*16 phage obtained by tRNAscan-SE. The tRNA type, length, genome position, and anticodon (marked square) are presented.


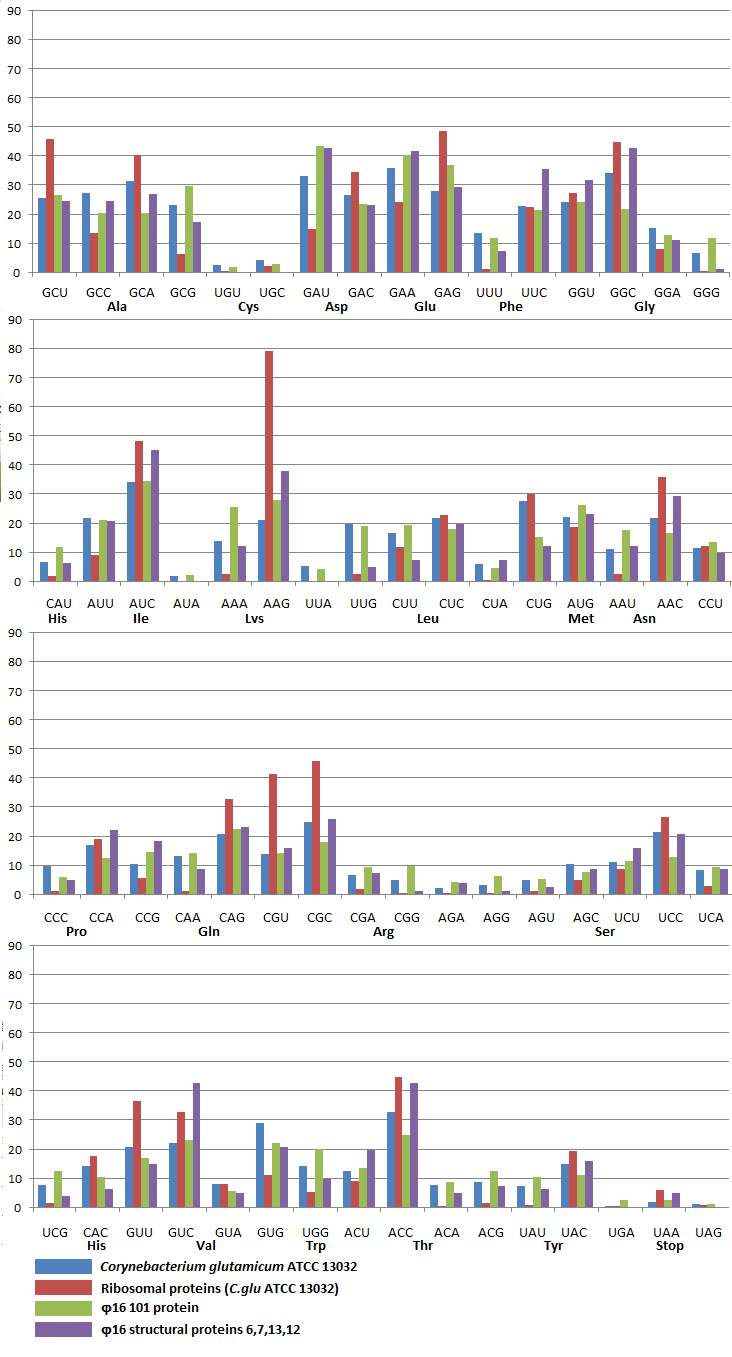


**Supplementary Fig. 3** Codon usage analysis was performed using the Countcodon program [ver. 4] (<http://www.kazusa.or.jp/codon/countcodon.html>)[[2]](#footnote-3). Codon usage numbers calculated for *Corynebacterium glutamicum* ATCC 13032 genes of the whole genome (blue), for ribosomal protein genes of *C. glutamicum* only (red), for *φ*16 genes of the whole genome (green) and for four structural genes of *φ*16 (purple).


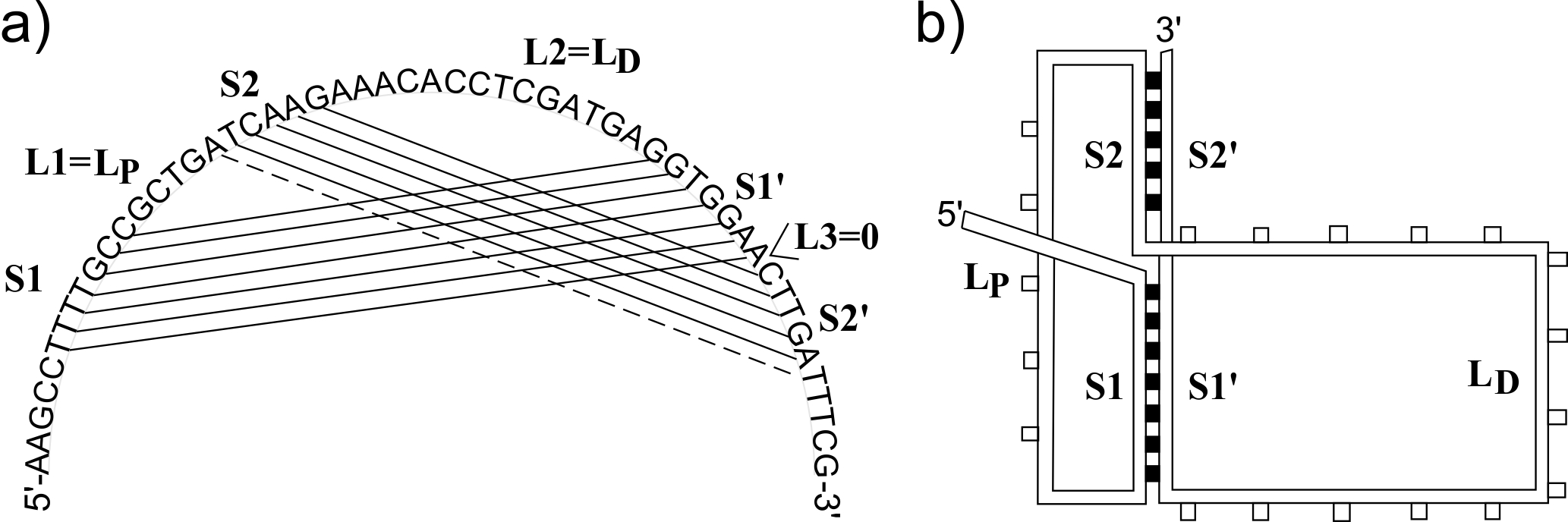


**Supplementary Fig. 4** The H-type pseudoknot secondary structure presentations of the generalized H-pseudoknot. The class of the H-type pseudoknot when connecting loop L3 is zero. An illustration of the Tinoco’s matrix building principles is shown on the left-hand side (a). The proposed base-pairing in the pseudoknot is indicated by thin lines. The structures on the right-hand side give a coaxial stacking of S1 and S2. L_D_ is the loop crossing the deep groove and Lp is the loop spanning the sugar-phosphate backbone (b). It should be noted that L_D_ = 14 nt is significantly larger than the minimal size, 2 nt, essential for bridging the deep groove of the quasi-continuous estimated double helix[[3]](#footnote-4), but L_P_ = 5 is the minimal length that, according to Monte Carlo-based calculations[[4]](#footnote-5), could be almost sufficient for the proposed stem crossing in the pseudoknot structure with coaxial stacking. The latter explains why an additional A-U (marked by the dashed line) is not included in the potential stem.


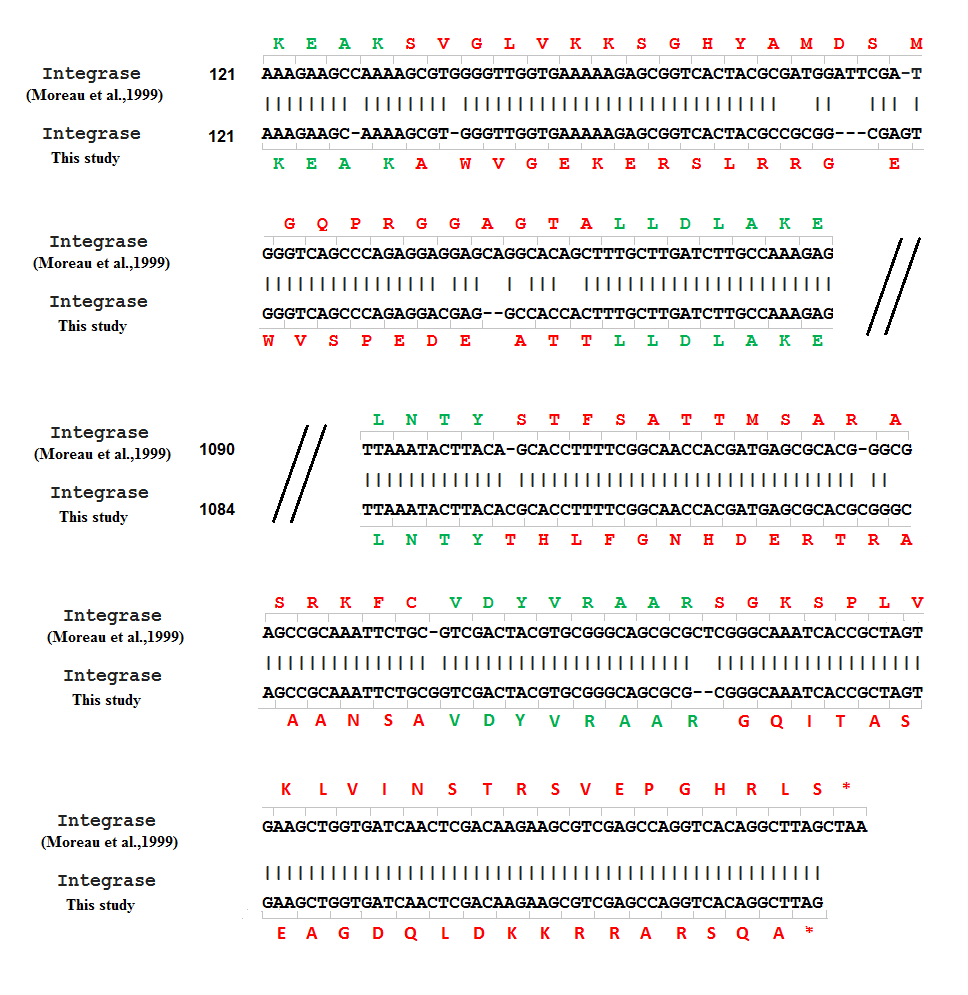


**Supplementary Fig. 5** Part of the nucleotide alignment of the *φ*16 integrase gene nucleotide sequence (GenBank:Y12471.1) previously deposited by Dr. Trautwetter’s group[[5]](#footnote-6) and the *φ*16 integrase gene ORF33 nucleotide sequence obtained in this work. Both nucleotide sequences are translated into amino acid sequences. Identical regions of amino acid sequences (green) and areas of mismatch (red) are shown.


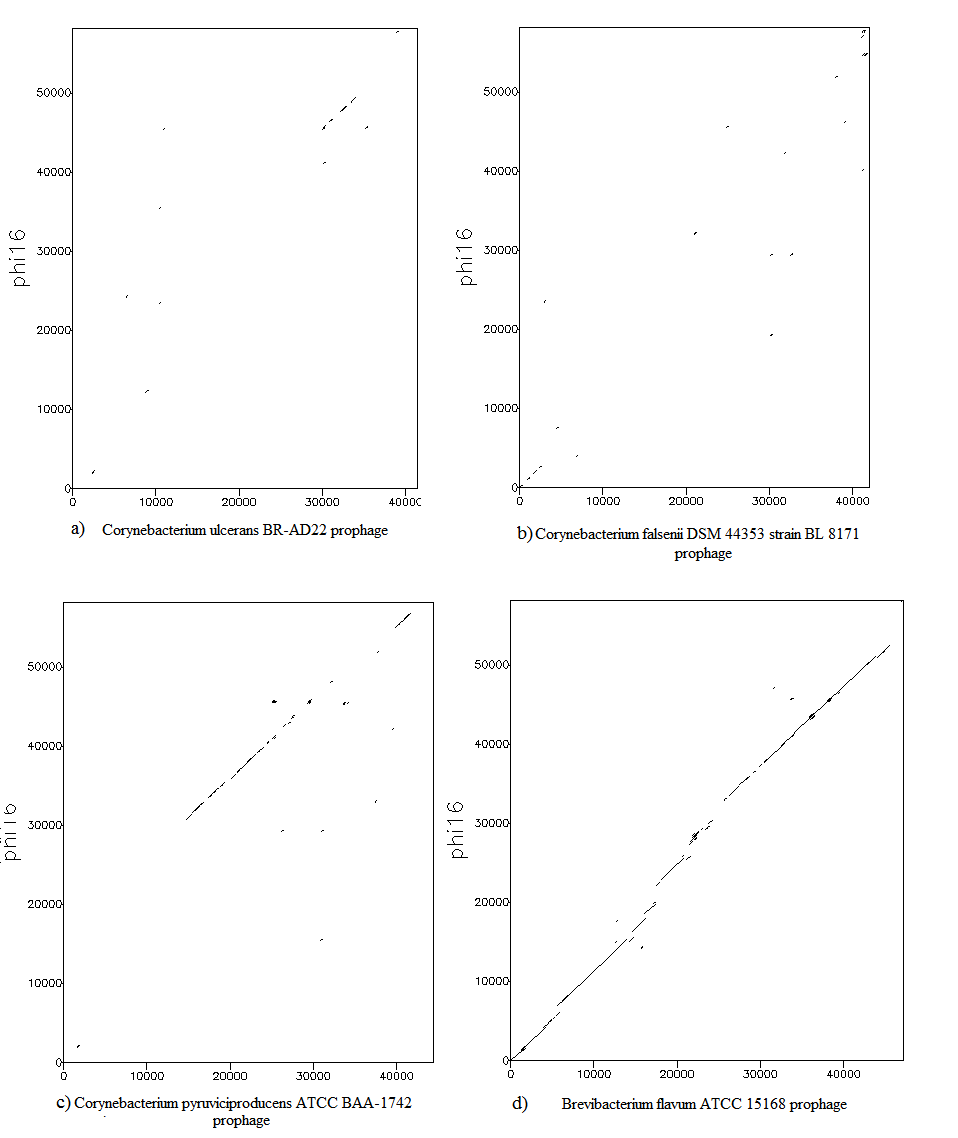


**Supplementary Fig. 6** DotPlot of *φ*16 genome sequence and related prophage sequences. DotPlots were constructed with the Dotmatcher tool from the EMBOSS package (<http://emboss.bioinformatics.nl/cgi-bin/emboss/dotmatcher>). A window size of 150 and a threshold value of 50 were used as parameters. For analysis, the whole prophage genome sequence of *φ*16 was used together with probable prophage sequences extracted from bacterial *Corynebacterium ulcerans* BR-AD22 (EMBL Acc CP002791.1) (**a**), *Corynebacterium falsenii* DSM 44353 strain BL 8171 (EMBL Acc CP007156.1) (**b**), *Corynebacterium pyruviciproducens* ATCC BAA-1742 (EMBL Acc ATBY00000000) (**c**) and *Brevibacterium flavum* ATCC 15168 (EMBL Acc CP011309.1) (identical to a prophage of *C. glutamicum* ATCC 14067 EMBL Acc AGQQ00000000) (**d**). DotPlots were constructed with the Dotmatcher tool (http://emboss.bioinformatics.nl/cgi-bin/emboss/dotmatcher). A window size of 150 and a threshold value of 50 were used as parameters.

**Supplementary Table 1** *φ*16 ORFs, gene products, and functional assignments

| ORF Start Stop length Strand RBS(spaser) Predicted Function Related proteins GenBank accession Superfamily E value Identity  (nt) | | | | | | | | | | | |
| --- | --- | --- | --- | --- | --- | --- | --- | --- | --- | --- | --- |
| 1 | 204 | 1076 | 873 | + | GAAAG 9 |  | hypothetical protein [*Corynebacterium diphtheriae*] | WP_045143446.1 |  | 3,00E-78 | 90% |
| 2 | 1149 | 1379 | 231 | + | AGGAG 7 | **Phage terminase,**  **small subunit*** | hypothetical protein [*Corynebacterium diphtheriae*] | WP_045143445.1 |  | 0,002 | 52% |
| 3 | 1495 | 3072 | 1578 | + | AGGAG 2 | **Phage terminase,**  **large subunit** | putative phage terminase, large subunit [*Corynebacterium jeikeium* ATCC 43734]  putative phage terminase, large subunit [*Rhodococcus equi* ATCC 33707]  Terminase [*Rhodococcus rhodochrous* ATCC 21198] | EEW17398.1  EGD24664.1  ETT26254.1 | Terminase_1_  superfamily | 2,00E-111  2,00E-45  2,00E-43 | 46%  29%  30% |
| 4 | 3082 | 4407 | 1326 | + | AGGAG(6) | **Portal protein** | portal protein [*Mycobacterium* phage Donovan]  portal protein [*Mycobacterium* phage Fishburne] | YP_009004374.1  YP_008051228.1 | Phage_portal,  DUF935  superfamily | 2,00E-85  1,00E-84 | 43%  43% |
| 5 | 4404 | 5567 | 1164 | + | AGGAG 8 | **Head maturation protease (capsid maturation protease)** | Clp protease [*Glaciibacter superstes*]  capsid maturation protease [*Gordonia* phage Blueberry]  ATP-dependent protease ClpP, protease subunit [*Gordonia westfalica*]  Clp protease [*Rhodococcus equi*]  capsid maturation protease [*Gordonia* phage CarolAnn] | WP_022886368.1  YP_009276629.1  SDU64683.1  WP_005513022.1  YP_009291959.1 | Crotonase-like_  Superfamily  Mu-like_Pro-like_  superfamily | 9E-65  2E-62  3E-61  5E-61  6E-61 | 40%  39%  38%  41%  38% |
| 6 | 5580 | 5978 | 399 | + | AGGAG 8 | **Structural protein**** | hypothetical protein [*Corynebacterium sp*. HMSC28B08] | WP_070433665.1 |  | 2E-28 | 51% |
| 7 | 6000 | 6944 | 945 | + | GAGG 6 | **Main capsid protein**  **(major capsid protein)**** | major capsid protein [*Gordonia* phage CaptainKirk2]  major capsid protein [*Gordonia* phage BetterKatz] | YP_009287223.1  YP_009302767.1 |  | 4E-41  2E-39 | 34%  35% |
| 8 | 6944 | 7306 | 363 | + | GAGG 6 |  | hypothetical protein [*Corynebacterium jeikeium*] | WP_049171420.1 |  | 4E-7 | 30% |
| 9 | 7330 | 7764 | 435 | + | GAGG 9 |  | hypothetical protein KIQ )15790 [*Corynebacterium glutamicum* ATCC 14067]  hypothetical protein [*Corynebacterium ulcerans*] hypothetical protein [*Corynebacterium jeikeium*] | KEI23960.1  WP_014525793.1WP_005293473.1 | Phage_Gp19_  superfamily | 1E-42  2E-24  2E-24 | 52%  43%  45% |
| 10 | 7761 | 8093 | 333 | + | AAGG 8 |  | hypothetical protein KIQ_015785 [*Corynebacterium glutamicum* ATCC 14067]  hypothetical protein [*Corynebacterium pyruviciproducens*] | KEI23959.1  WP_016457085.1 |  | 2,00E-31  2,00E-15 | 52%  39% |
| 11 | 8093 | 8392 | 300 | + | GGAG 5 |  | hypothetical protein KIQ_015780 [*Corynebacterium glutamicum* ATCC 14067]  hypothetical protein [*Corynebacterium pyruviciproducens*] | KEI23958.1  WP_016457086.1 | TraC_F_IV  superfamilly | 3,00E-36  1,00E-11 | 63%  39% |
| 12 | 8392 | 8805 | 414 | + | AGGA 6 | **Structural protein**** | hypothetical protein KIQ_015775[*Corynebacterium glutamicum* ATCC 14067]  hypothetical protein [*Corynebacterium pyruviciproducens*] | KEI23957.1  WP_016457087.1 |  | 5,00E-57  1,00E-15 | 65%  36% |
| 13 | 8789 | 9484 | 696 | + | AAGG 9 | **Major tail protein**** | hypothetical protein KIQ_015770 [*Corynebacterium glutamicum* ATCC 14067]  hypothetical protein [*Corynebacterium pyruviciproducens*] | KEI23956.1  WP_016457088.1 |  | 8,00E-118  1,00E-50 | 77%  42% |
| 14 | 9613 | 9978 | 366 | + | AGGA 9 |  | hypothetical protein KIQ_015765 [*Corynebacterium glutamicum* ATCC 14067]  hypothetical protein [*Corynebacterium ulcerans*] | KEI23955.1  WP_014836406.1 |  | 7,00E-75  5,00E-15 | 95%  42% |
| 15 | 10014 | 10340 | 327 | + | ND |  | hypothetical protein KIQ_015760 [*Corynebacterium glutamicum* ATCC 14067]  hypothetical protein [*Corynebacterium ulcerans*] | KEI23954.1  WP_013911529.1 |  | 5,00E-51  6,00E-18 | 83%  47% |
| 16 | 10387 | 16842 | 6456 | + | GGAGG 10 | **Phage tail tape measure protein**  **(minor tail protein)** | [phage tail tape measure protein, TP901 family, immunity-specific protein beta241[](http://blast.ncbi.nlm.nih.gov/Blast.cgi#alnHdr_258604143)*Corynebacterium jeikeium* ATCC 43734]  phage tail tape measure protein [*Corynebacterium ulcerans*]  phage tail tape measure protein [*Gordonia soli*]  tape measure protein [*Rhodococcus* phage ChewyVIII] | WP_005293452.1  WP_032802857.1  WP_007625661.1  AON97476.1 | Tape_meas_nterm_  superfamily | 2,00E-126  1,00E-97  1,00E-68  1E-49 | 28%  29%  29%  33% |
| 17 | 16852 | 17619 | 768 | + | GGAGG 5 |  | hypothetical protein,Immunity-specific protein beta201 [*Corynebacterium diphtheriae* strain 31A] | WP_014316207.1 |  | 1,00E-22 | 31% |
| 18 | 17623 | 18507 | 885 | + | GGAGG 6 |  | immunity-specific protein Beta286 [*Corynebacterium diphtheriae* C7 (beta)] | WP_014318500.1 |  | 2,00E-44 | 37% |
| 19 | 18509 | 19348 | 840 | + | AGGAG 9 |  | hypothetical protein KIQ_015740 [*Corynebacterium glutamicum* ATCC 14067]  immunity-specific protein Beta371 [*Corynebacterium diphtheriae* 31A] | KEI23952.1  AEX40899.1 |  | 0  1E-37 | 97%  42% |
| 20 | 19360 | 20547 | 1188 | + | GGAGG 7 | **Phage tail fiber protein** | phage tail fiber protein [*Corynebacterium diphtheriae* NCTC 13129] | NP_938609.1 |  | 4,00E-07 | 25% |
| 21 | 20544 | 20888 | 345 | + | ND |  | hypothetical protein KIQ_015725 [*Corynebacterium glutamicum* ATCC 14067] | KEI23949.1 |  | 2,00E-43 | 76% |
| 22 | 20936 | 22357 | 1422 | + | AGGAG 8 | **Lysin** | glycosyl hydrolase family 25 [*Corynebacterium glutamicum* ATCC 14067]  LysA [*Rhodococcus* phage ReqiPine5]  lysin [*Rhodococcus* phage RGL3] | KEI23948.1  YP_009016221.1  YP_005087024.1 | Peptidase_M23 superfamily  GH25_muramidase superfamily | 3,00E-147  1,00E-101  2,00E-75 | 52%  45%  39% |
| 23 | 22347 | 22589 | 243 | + | AGGAGG 6 | **Holin** | holin [*Corynebacterium pseudotuberculosis*]  holin [*Corynebacterium ulcerans*]  holin [*Corynebacterium diphtheriae*]  holin [*Mycobacterium* phage BuzzLyseyear] | WP_048653288.1  WP_014835770.1  WP_014306321.1  AIM50157.1 | Phage_r1t_holin superfamily  BCS1_N  superfamily | 3,00E-32  2,00E-30  3,00E-27  9,00E-10 | 73%  70%  67%  48% |
| 24 | 22589 | 23095 | 507 | + | ND |  | ND |  |  |  |  |
| 25 | 23623 | 23117 | 507 | - | GAGG 7 |  | ND |  |  |  |  |
| 26 | 24170 | 23841 | 330 | - | AGGA 5 |  | ND |  |  |  |  |
| 27 | 24498 | 24199 | 300 | - | AAGG 8 |  | ND |  |  |  |  |
| 28 | 25491 | 24604 | 888 | - | ND | **Integrase** | site-spesific recombinase XerD [*Corynebacterium timonense*]  Uncharacterized protein *Corynebacterium pyruviciproducens* ATCC BAA-1742  Phage integrase SAM-like domain protein *Bifidobacterium pseudocatenulatum* CAG:263  integrase [*Gordonia* phage Utz] | WP_019193356.1  WP_016457103.1  [WP_022244954.1](http://www.ncbi.nlm.nih.gov/protein/547837188?report=genbank&log$=prottop&blast_rank=2&RID=VACP56MX01R)  YP_009274976.1 | DNA_BRE_C  superfamily | 3,00E-110  1,00E-103  1.3e-39  7,00E-22 | 58%  55%  36%  31% |
| 29 | 26357 | 25479 | 879 | - | GAAAG 10 |  | hypothetical protein [*Corynebacterium timonense*]  hypothetical protein HMPREF1219_00180 [*Corynebacterium pyruviciproducens* ATCC BAA-1742] | WP_019193357.1  WP_016457104.1 |  | 4,00E-107  6,00E-79 | 57%  46% |
| 30 | 27037 | 26360 | 678 | - | AGGA 11 |  | hypothetical protein [*Corynebacterium sp*. JZ16]  hypothetical protein CFAL_09590 [*Corynebacterium falsenii* DSM 44353]  hypothetical protein [*Corynebacterium timonense*] | WP_066570128.1  AHI04359.1  WP_019193358.1 |  | 8,00E-53  3,00E-07  4,00E-06 | 49%  27%  34% |
| 31 | 27386 | 27099 | 288 | - | AAGG 8 |  | ND |  |  |  |  |
| 32 | 27709 | 27563 | 147 | - | ND |  | ND |  |  |  |  |
| 33 | 29084 | 27843 | 1242 | - | AGGGA 11 | **Integrase***** | intergrase [Corynephage phi16]  integrase [Corynephage 304L]  hypothetical protein CFAL_09875 [*Corynebacterium falsenii* DSM 44353] | CAA73074.1  CAB38562.1  AHI04398.1 | DNA_BRE_C s  uperfamily  Phage_int_SAM_4 superfamily | 0.0  1E-109  3E-53 | 90%  47%  36% |
| 34 | 29745 | 29203 | 543 | - | AGGA 8 | **Phage super infection exclusion** | phage super infection exclusion [*Corynebacterium accolens*]  MULTISPECIES: phage super infection exclusion [*Corynebacterium*] | WP_039885942.1  WP_034653861.1 | Lipoprotein_Ltp superfamily | 8,00E-50  1,00E-48 | 52%  52% |
| 35 | 30133 | 29759 | 375 | - | GGAGG 4 | **Metallo-endopeptidase** | MULTISPECIES: ImmA/IrrE family metallo-endopeptidase [*Corynebacterium*]  hypothetical protein CFAL_09865 [*Corynebacterium falsenii* DSM 44353] | WP_046552195.1  AHI03845.1 | DUF955  superfamily | 6,00E-84  1,00E-13 | 99%  38% |
| 36 | 30700 | 30143 | 570 | - | ND | **DNA-binding protein (transcriptional regulators)** | transcriptional regulator [Corynebacterium glutamicum]  transcriptional regulator [Gordonia sp. QH-12]  hypothetical protein [Corynebacterium pyruviciproducens] | WP_063967696.1  WP_062367315.1  WP_016457043.1 | HTH_XRE  superfamily | 5E-134  5E-27  3,00E-06 | 100%  47% 32% |
| 37 | 30825 | 31070 | 246 | + | GGAGG 9 |  | MULTISPECIES: hypothetical protein [*Corynebacterium*] | WP_040072914.1 | CPSase_L_D3 superfamily | 2,00E-51 | 99% |
| 38 | 31103 | 31348 | 246 | + | GGAGG 8 |  | MULTISPECIES: hypothetical protein [*Corynebacterium*] | WP_040072913.1 |  | 3,00E-52 | 100% |
| 39 | 31402 | 31551 | 150 | + | ND |  | MULTISPECIES: hypothetical protein [*Corynebacterium*] | WP_040072912.1 |  | 4,00E-26 | 96% |
| 40 | 31618 | 31866 | 249 | + | AAGG 10 |  | MULTISPECIES: hypothetical protein [*Corynebacterium*] | WP_040072911.1 |  | 9,00E-52 | 96% |
| 41 | 31884 | 32123 | 240 | + | AGGAG 11 |  | ND |  |  |  |  |
| 42 | 32107 | 33042 | 936 | + | GAGG 6 | **ParB-like nuclease domain protein** | ParB-like protein [*Corynebacterium jeikeium* ATCC 43734] | WP_005293568.1 | ParBc  superfamily | 2,00E-24 | 33% |
| 43 | 33018 | 33581 | 564 | + | AGGA 6 |  | hypothetical protein HMPREF0297_0301 [*Corynebacterium jeikeium* ATCC 43734] | WP_005293566.1 |  | 6,00E-06 | 29% |
| 44 | 33954 | 34889 | 936 | + | AGGA 6 |  | hypothetical protein MA5S0422_3968 [*Mycobacterium abscessus* 5S-0422  gp50 [*Mycobacterium* phage Mutaforma13] | EIU03872.1  AEJ93129.1 |  | 2,00E-65  3,00E-26 | 41%  33% |
| 45 | 34958 | 35098 | 141 | + | GGAGG 8 |  | ND |  |  |  |  |
| 46 | 35095 | 35295 | 201 | + | AGGAG 8 |  | MULTISPECIES: hypothetical protein [*Corynebacterium*] | WP_040072908.1 |  | 3E-23 | 67% |
| 47 | 35288 | 35467 | 180 | + | GGGGA 6 | **Excisionase/Xis***** | MULTISPECIES: helix-turn-helix domain-containing protein [*Corynebacterium*]  hypothetical protein CFAL_09845 [*Corynebacterium falsenii DSM* 44353]  excisionase/Xis, DNA-binding protein [*Corynebacterium jeikeium* ATCC 43734] | WP_040072907.1  AHI04396.1  WP_005293561.1 | HTH_MerR-SF superfamily  DNA binding domain, excisionase family | 9,00E-29  8,00E-06  1,00E-04 | 86%  40%  37% |
| 48 | 35491 | 35859 | 369 | + | GGAGG 5 |  | MULTISPECIES: hypothetical protein [*Corynebacterium*]  hypothetical protein [*Rhodococcus sp*. P27] | WP_044394326.1  WP_021343820.1 | DUF 2303 | 8,00E-51  3,00E-24 | 66%  45% |
| 49 | 35874 | 36734 | 861 | + | AGGA 10 |  | MULTISPECIES: hypothetical protein [*Corynebacterium*]  hypothetical protein [*Rhodococcus sp*. P27] | WP_040072906.1  WP_021343819.1 | DUF2303  superfamily | 6,00E-141  3,00E-50 | 68%  35% |
| 50 | 36725 | 36907 | 183 | + | GAAG 10 |  | MULTISPECIES: hypothetical protein [*Corynebacterium*] | WP_040072905.1 |  | 4,00E-31 | 97% |
| 51 | 36894 | 37271 | 378 | + | ND |  | hypothetical protein KIQ_016025 [*Corynebacterium glutamicum* ATCC 14067]  unnamed protein product [*Mycobacterium* phage Faith1] | KEI24001.1  YP_004539010.1 |  | 2,00E-56  8,00E-04 | 97%  32% |
| 52 | 37268 | 37639 | 372 | + | GGAG 6 | **HNH endonuclease domain protein** | hypothetical protein KIQ_016020 [*Corynebacterium glutamicum* ATCC 14067]  HNH endonuclease domain protein [*Corynebacterium jeikeium* ATCC 43734] | KEI24000.1  EEW17414.1 | DUF3268  superfamily | 1,00E-84  1,00E-12 | 99%  38% |
| 53 | 37632 | 38045 | 414 | + | GAAG 6 |  | hypothetical protein KIQ_016015 [*Corynebacterium glutamicum* ATCC 14067] | KEI23999.1 |  | 8,00E-89 | 95% |
| 54 | 38056 | 38187 | 132 | + | GGAGG 6 |  | hypothetical protein KIQ_00080 [*Corynebacterium glutamicum* ATCC 14067] | **EHE84798.1** | SAM_  superfamily | 5,00E-19 | 91% |
| 55 | 38184 | 38516 | 333 | + | GGAGG 4 |  | hypothetical protein KIQ_016005 [*Corynebacterium glutamicum* ATCC 14067] | KEI23998.1 |  | 4,00E-60 | 99% |
| 56 | 38533 | 39390 | 858 | + | GGAGG 5 | **Transcriptional regulator** | HTH-type transcriptional regulator KIQ_00090 [*Corynebacterium glutamicum* ATCC 14067]  HTH DNA binding protein [*Mycobacterium* phage Fishburne] | EHE84800.1  AGM12949.1 | HTH_36  superfamily | 3,00E-167  7,00E-21 | 98%  54% |
| 57 | 39400 | 39909 | 510 | + | GGAGG 5 |  | hypothetical protein KIQ_015995 [*Corynebacterium glutamicum* ATCC 14067] | KEI23996.1 | Glyco_hydro_1 superfamily | 1,00E-119 | 99% |
| 58 | 39910 | 40470 | 561 | + | AGGA 9 | **SSB_OBF Single-stranded DNA-binding protein** | single-stranded DNA-binding protein [*Corynebacterium glutamicum* ATCC 14067] | KEI23995.1 | RPA_2b-aaRSs_  OBF_like  superfamily | 2,00E-115 | 95% |
| 59 | 40480 | 40746 | 267 | + | GGAG 5 |  | hypothetical protein KIQ_015985 [*Corynebacterium glutamicum* ATCC 14067] | KEI23994.1 |  | 4,00E-50 | 88% |
| 60 | 40743 | 41069 | 327 | + | AGGA 7 |  | hypothetical protein KIQ_015980[*Corynebacterium glutamicum* ATCC 14067] | KEI23993.1 |  | 3,00E-69 | 91% |
| 61 | 41114 | 41509 | 396 | + | AGGAG 5 |  | hypothetical protein KIQ_015975[*Corynebacterium glutamicum* ATCC 14067] | KEI23992.1 |  | 2,00E-61 | 73% |
| 62 | 41502 | 41765 | 264 | + | AGGA 11 | **Glutaredoxin-like protein** | hypothetical protein KIQ_015970 [*Corynebacterium glutamicum* ATCC 14067]  gp70 [*Mycobacterium* phage fionn] | KEI23991.1  YP_009150934.1 | Thioredoxin_like superfamily | 2,00E-42  1,00E-07 | 97%  35% |
|  |  |  |  |  |  |  | glutaredoxin [*Rhodococcus opacus*] | WP_005254985.1 |  | 2,00E-07 | 36% |
| 63 | 41765 | 42238 | 474 | + | AAGG 10 | **Crossover junction**  **Endodeoxyribonucle- ase RusA** | hypothetical protein KIQ_015965[*Corynebacterium glutamicum* ATCC 14067]  resolvase [*Gordonia* phage BritBrat]  RusA [*Mycobacterium* phage Sbash]  crossover junction endodeoxyribonuclease RusA [*Rhodococcus equi* ATCC 33707] | KEI23990.1  YP_009276616.1  AJA43362.1  EGD23951.1 |  | 1,00E-92  4,00E-42  2,00E-13  3,00E-08 | 83%  55%  37%  37% |
| 64 | 42262 | 42732 | 471 | + | GGAGG 8 | **Methyltransferase** | methyltransferase [*Corynebacterium glutamicum* ATCC 14067] | KEI23989.1 | AdoMet_Mtases superfamily | 9,00E-103 | 93% |
| 65 | 42729 | 43010 | 282 | + | GGAGG 4 |  | hypothetical protein KIQ_015955 [*Corynebacterium glutamicum* ATCC 14067] | KEI23988.1 |  | 1,00E-42 | 75% |
| 66 | 43007 | 43306 | 300 | + | GGAGG 4 |  | hypothetical protein KIQ_015945 [*Corynebacterium glutamicum* ATCC 14067] | KEI23986.1 |  | 8,00E-44 | 69% |
| 67 | 43303 | 44220 | 918 | + | AGGA 5 |  | hypothetical protein [*Corynebacterium glutamicum*] | WP_044027492.1 |  | 8E-39 | 38% |
| 68 | 44217 | 44930 | 714 | + | GGAG 7 | **Chromosome segregation protein SMC** | hypothetical protein KIQ_015940 [*Corynebacterium glutamicum* ATCC 14067] | KEI23985.1 | Chromosome_partition_protein_Smc,  DUF3584,COG4942 | 9,00E-96 | 63% |
| 69 | 44917 | 45270 | 354 | + | GAGG 9 |  | hypothetical protein KIQ_015935 [*Corynebacterium glutamicum* ATCC 14067] | KEI23984.1 | DUF4406 TIR_2 superfamily | 6,00E-80 | 98% |
|  |  |  |  |  |  |  | gp42 [*Corynebacterium* phage BFK20]  CMP N-glycosidase [*Arthrobacter* phage Galaxy] | YP_001456772.1  ALY08894.1 |  | 8,00E-20  7,00E-22 | 45%  44% |
| 70 | 45257 | 45685 | 429 | + | GGAGG 5 |  | hypothetical protein KIQ_015930 [*Corynebacterium glutamicum* ATCC 14067]  gp68 [*Mycobacterium* phage Alma] | KEI23983.1  YP_009014933.1 |  | 4,00E-96  6,00E-30 | 99%  46% |
| 71 | 45682 | 45864 | 183 | + | GGAGG 10 |  | ND |  |  |  |  |
| 72 | 45851 | 46117 | 267 | + | GGAGG 6 |  | ND |  |  |  |  |
| 73 | 46114 | 46488 | 375 | + | AAGG 9 |  | hypothetical protein KIQ_013605 [*Corynebacterium glutamicum* ATCC 14067] | KEI23557.1 |  | 4,00E-08 | 46% |
| 74 | 46478 | 46708 | 231 | + | GGAGG 3 |  | ND |  |  |  |  |
| 75 | 46701 | 47033 | 333 | + | ND |  | hypothetical protein KIQ_00170 [*Corynebacterium glutamicum* ATCC 14067] | EHE84816.1 |  | 2,00E-30 | 71% |
| 76 | 47033 | 47236 | 204 | + | GGAGG 7 |  | hypothetical protein KIQ_00175 [*Corynebacterium glutamicum* ATCC 14067] | EHE84817.1 |  | 6,00E-36 | 93% |
| 77 | 47208 | 47471 | 264 | + | GGAG 7 |  | hypothetical protein KIQ_00180 [*Corynebacterium glutamicum* ATCC 14067] | EHE84818.1 |  | 2,00E-43 | 89% |
| 78 | 47468 | 47695 | 228 | + | GGAGG 7 |  | ND |  |  |  |  |
| 79 | 47806 | 48291 | 486 | + | GGAGG 4 |  | hypothetical protein [*Mycobacterium conceptionense*]  unnamed protein product [Mycobacterium phage Wee] | WP_048895601.1  YP_004123908.1 |  | 2,00E-67  8,00E-64 | 68%  67% |
| 80 | 48284 | 48559 | 276 | + | AGGAG 7 |  | ND |  |  |  |  |
| 81 | 48550 | 48798 | 249 | + | AGGAG 5 |  | ND |  |  |  |  |
| 82 | 48795 | 48974 | 180 | + | GGAG 5 |  | ND |  |  |  |  |
| 83 | 48971 | 49195 | 225 | + | GGAG 5 |  | ND |  |  |  |  |
| 84 | 49188 | 49409 | 222 | + | GGAGG 6 |  | ND |  |  |  |  |
| 85 | 49410 | 49634 | 225 | + | ND |  | ND |  |  |  |  |
| 86 | 49648 | 50058 | 411 | + | AGGA 6 |  | hypothetical protein KIQ_013630 [*Corynebacterium glutamicum* ATCC 14067] | KEI23562.1 |  | 2,00E-07 | 31% |
| 87 | 50051 | 50227 | 177 | + | GGAGG 6 |  | hypothetical protein KIQ_00190 [*Corynebacterium glutamicum* ATCC 14067] | EHE84820.1. |  | 2,00E-30 | 86% |
| 88 | 50220 | 50510 | 291 | + | GAGG 7 |  | hypothetical protein KIQ_013625 [*Corynebacterium glutamicum* ATCC 14067] | KEI23561.1 |  | 5,00E-10 | 83% |
| 89 | 50507 | 50815 | 309 | + | GGAG 9 |  | ND |  |  |  |  |
| 90 | 50812 | 51189 | 378 | + | GGAGG 7 |  | hypothetical protein KIQ_015895 [*Corynebacterium glutamicum* ATCC 14067] | KEI23977.1 |  | 1,00E-68 | 81% |
| 91 | 51162 | 51434 | 273 | + | GGAGG 7 |  | hypothetical protein KIQ_015890 [*Corynebacterium glutamicum* ATCC 14067] | KEI23976.1 |  | 5,00E-33 | 69% |
| 92 | 51709 | 51930 | 222 | + | AGGA 8 |  | hypothetical protein KIQ_015885 [*Corynebacterium glutamicum* ATCC 14067 | KEI23975.1 | DUF3991,  DUF 4614 superfamily | 1,00E-37 | 97% |
| 93 | 51911 | 52225 | 315 | + | ND |  | hypothetical protein KIQ_015880 [*Corynebacterium glutamicum* ATCC 14067] | KEI23974.1 |  | 2,00E-32 | 57% |
| 94 | 52222 | 52860 | 639 | + | GAGG 5 | **Oligoribonuclease** | oligoribonuclease [*Corynebacterium pyruviciproducens*]  exonuclease [*Gordonia* phage Jumbo]  exonuclease [*Tsukamurella* phage TIN3] | WP_016457995.1  YP_009291020.1  YP_009214815.1 | DnaQ_like_exo superfamily | 2,00E-19  1,00E-18  3,00E-17 | 37%  37%  34% |
| 95 | 53065 | 53307 | 243 | + | ND |  | hypothetical protein KIQ_015870 [*Corynebacterium glutamicum* ATCC 14067] | KEI23972.1 |  | 2,00E-43 | 90% |
| 96 | 53310 | 53555 | 246 | + | GGAGG 8 |  | hypothetical protein KIQ_015860 [*Corynebacterium glutamicum* ATCC 14067] | KEI23970.1 |  | 1,00E-44 | 89% |
| 97 | 53773 | 54396 | 624 | + | ND |  | hypothetical protein HMPREF1261_02242 [*Corynebacterium sp*. KPL1818] | WP_023030988.1 |  | 2,00E-27 | 37% |
| 98 | 54464 | 55177 | 714 | + | GGAGG 5 |  | hypothetical protein [*Mycobacterium chelonae*] | WP_070930292.1 |  | 2,00E-29 | 37% |
| 99 | 56438 | 56758 | 321 | + | AGGAG 8 |  | hypothetical protein KIQ_015845 [*Corynebacterium glutamicum* ATCC 14067] | KEI23969.1 |  | 1,00E-15 | 36% |
| 100 | 56742 | 56990 | 249 | + | GGAGG 9 |  | hypothetical protein KIQ_013580 [*Corynebacterium glutamicum* ATCC 14067] | KEI23552.1 |  | 4,00E-04 | 32% |
| 101 | 57024 | 58118 | 1095 | + | GGAGG 6 | **ATPase** | ATPase AAA [*Corynebacterium efficiens*]  gp82 [*Mycobacterium* phage BigNuz] | WP_006769474.1  YP_009012331.1 | ATP_bind_2,  P-loop_NTPase Superfamily | 1,00E-42  8,00E-31 | 32%  48% |

*Function is predicted based on mutual arrangement

**Structural proteins detected by SDS-PAGE

***Predicted function is experimentally confirmed

**Supplementary Table 2** Putative promoters. Nucleotide sequences of predicted putative promoter “-10” and “-35” sequences are highlighted.

| Promoter | 5` -35 -10 3` |
| --- | --- |
| P_R1_ | TGGTCGAAAAGTGGAA-AAACGTGCTCTGACCTGCTAAAATAGAGTGAAAG |
| P_R2_ | AGGATCTTCATTGAAA---GGAATCACCACTATGGCAAAGTCCATCGATAA |
| P_R3_ | ACCATATGAGTTGACA--AGTTTCAGCGCTTGGTGCATACTTACATCAGTG |
| P_R4_ | AAAGAAAAGCTTGAAAAAGCTGTTGAGTGGGCCAATATGGTGGTCAATCCG |
| P_R5_ | GCGGAGCTGCTTGACATCAAGGTTATTAATCCAAATATTTTAGGAGGTTCT |
| P_R6_ | AGTATCCCGATACGCA-ACACTCCATCGGCCTCACTACATTTGTCCCGAAT |
| P_L1_ | CATGCAGGTGTTGCAC--TATTTATTGAGTGATGATAAAATTCATCACATG |

**Supplementary Table 3** Putative intrinsic transcription terminators. Nucleotide sequences of predicted putative terminators, the termination loop (red) and the hairpin (blue) are marked. Transcription terminators were folded using the RNAfold program (<http://rna.tbi.univie.ac.at/cgi-bin/RNAfold.cgi>)

| Terminator | Transcription terminators nucleotide sequence | ΔG, -kcal/mol |
| --- | --- | --- |
| T_R1_ | TGGAATTTGTAGACAGTCCTGGTGGGCTGTCTTTTTGTTTTCA | 13.8 |
| T_R2_ | AAGCTTTATAATCCCCGGCATGAGCTGGGGATTTTCTAATTAG | 15.2 |
| T_R3_ | CTAACACTCTTGCCCCCACTGACCTTTATTGGTCAGTGGGGGCTTTTCCTATTCTC | 30.9 |
| T_R4_ | AATTTTTTGACCCGATCCACCGAATGCAGTGGGTCGGTTTTGGAAGGAG | 14.7 |
| T_R5_ | TTGTACCCGATCGGTTTAGGGATTGTTTCTTAGGCCGATTTTTTTAGGCG | 12.8 |
| T_R6_ | CGATTTTTTTAGGCGCCGACTCGTTGTGGTCGGCGCCTTTTTGGATCCA | 20.7 |
| T_R7_ | CTTGAAAATCACCCCGAATCTTATAGTTCGGGGTTTTGCTATTCC | 12.4 |
| T_L1_ | ATCTCTAGCTTTCCCCGCATTCGCGGGGGTTGCTTTTCCGC | 13.2 |

1. Pfeifer-Sancar K, Mentz A, Ruckert C, Kalinowski J (2013) Comprehensive analysis of the Corynebacterium glutamicum transcriptome using an improved RNAseq technique. BMC Genomics 14: 888. doi: 10.1186/1471-2164-14-888 [↑](#footnote-ref-2)
2. Nakamura Y, Gojobori T, Ikemura T (2000) Codon usage tabulated from the international DNA sequence databases: status for the year 2000. Nucleic Acids Res 28:292 [↑](#footnote-ref-3)
3. Pleij CWA (1994) RNA pseudoknots. Curr Opin Struct Biol 4:337-344 [↑](#footnote-ref-4)
4. Giedroc DP, Theimer CA, Nixon PL (2000) Structure, stability and function of RNA pseudoknots involved in stimulating ribosomal frameshifting. J Mol Biol 298:167–185. doi:[10.1006/jmbi.2000.3668](https://dx.doi.org/10.1006/jmbi.2000.3668) [↑](#footnote-ref-5)
5. Moreau S, Blanco C, Trautwetter A (1999) Site-specific integration of corynephage φ16: Construction of an integration vector. Microbiology 145:539–548. doi: doi:10.1099/13500872-145-3-539 [↑](#footnote-ref-6)
